# Novel Approaches to Monitor Pharmacokinetics and Metabolism of Gemcitabine-Ibandronate Conjugate in Mice and Dogs

**DOI:** 10.3390/molecules30020354

**Published:** 2025-01-16

**Authors:** Jost Klawitter, Mckay Easton, Alexander Karpeisky, Kristen B. Farrell, Douglas H. Thamm, Touraj Shokati, Uwe Christians, Shawn Patrick Zinnen

**Affiliations:** 1 Department of Anesthesiology, School of Medicine, University of Colorado, Anschutz Medical Campus, Aurora, CO 80045, USA; mckay.easton@gmail.com (M.E.); touraj.shokati@hepquant.com (T.S.); uwe.christians@cuanschutz.edu (U.C.); 2 Department of Psychiatry, School of Medicine, University of Colorado, Anschutz Medical Campus, Aurora, CO 80045, USA; 3 MBC Pharma Inc., Aurora, CO 80045, USA; alkarp@mbcpharma.com (A.K.); szinnen@mbcpharma.com (S.P.Z.); 4 Flint Animal Cancer Center, Colorado State University, Fort Collins, CO 80523, USA; kristen.farrell@colostate.edu (K.B.F.); doug.thamm@colostate.edu (D.H.T.)

**Keywords:** bisphosphonates, bone cancer, pharmacokinetics, gemcitabine ibandronate, high-performance liquid chromatography, mass spectrometry, drug metabolism

## Abstract

Background. The use of the bone-seeking properties of bisphosphonates (BPs) to target the delivery of therapeutic drugs is a promising approach for the treatment of bone metastases. Currently, the most advanced example of this approach is a gemcitabine-ibandronate conjugate (GEM-IB), where the bone-targeting BP ibandronate (IB) is covalently linked to the antineoplastic agent gemcitabine (GEM) via a spacer phosphate group. In the present study, we describe the development of a new analytical platform to evaluate the metabolism and pharmacokinetics of GEM-IB in mice and dogs and the results of proof-of-concept studies assessing the pharmacokinetics of GEM-IB in dogs and mice. Methods. We validated analytical platforms to analyze GEM-IB and five of its major metabolites IB, gemcitabine-5′-phosphate (GEMMP), gemcitabine (GEM), 2′,2′-difluoro-2′-deoxyuridine-5′-phosphate (dFdUMP), and 2′,2′-difluoro-2′-deoxyuridine (dFdU) and performed proof-of-concept pharmacokinetic studies in mice (5 mg/kg i.p.) and dogs (5 mg/kg i.v.). Results. Intra- and inter-run accuracy and imprecision (3 days) of the assays met the (FDA) acceptance criteria. The proof-of-concept plasma pharmacokinetic studies in mice showed AUCs of 1278, 10,652, 405, 38, 1063, 3389, and 38 h·ng/mL for GEM-IB, IB, GEMMP, dFdU-MP, GEM, and dFdU, respectively. In dog plasma, AUCs of 295, 5725, 83, 11, 1625, and 6569 h·ng/mL were observed for GEM-IB, IB, GEMMP, dFdUMP, GEM, and dFdU. Conclusions. Pharmacokinetic studies in dogs and mice showed that GEM-IB is rapidly converted to IB and GEM; dFdU is formed (from GEM) with a delay. The rapid disappearance of GEM-IB from circulation could be explained by a combination of metabolism and rapid distribution to tissue/bone.

## 1. Introduction

Tumor-induced bone disease (TIBD) is one of the major causes of morbidity in cancer patients [1,2]. More than half of all carcinoma patients develop bone metastases [3,4]. TIBD is found in 65–95% of patients with multiple myeloma and advanced breast and prostate cancers [2,5]. Because TIBD is associated with considerable morbidity and a median survival time of less than two years [2], the development of more effective therapies is warranted [5,6]. Current TIBD therapies are palliative. Standard anti-cancer chemotherapies at their maximum tolerated doses are unable to reach effective concentrations in the bone and its microenvironment. With the bone microenvironment and metastases to this site being critical to cancer progression [7], there is clearly a need for drugs that can attack and kill bone-associated tumor cells without prohibitive systemic toxicity.

Bisphosphonates (BPs) are bone-specific palliative treatments that reduce tumor-induced skeletal complications [1,8,9,10]. However, TIBD still progresses in BP-treated cancer patients. The development of drugs with enhanced anti-resorptive and cytotoxic characteristics to improve the treatment of patients with TIBD is greatly needed [1].

**Figure 1:**
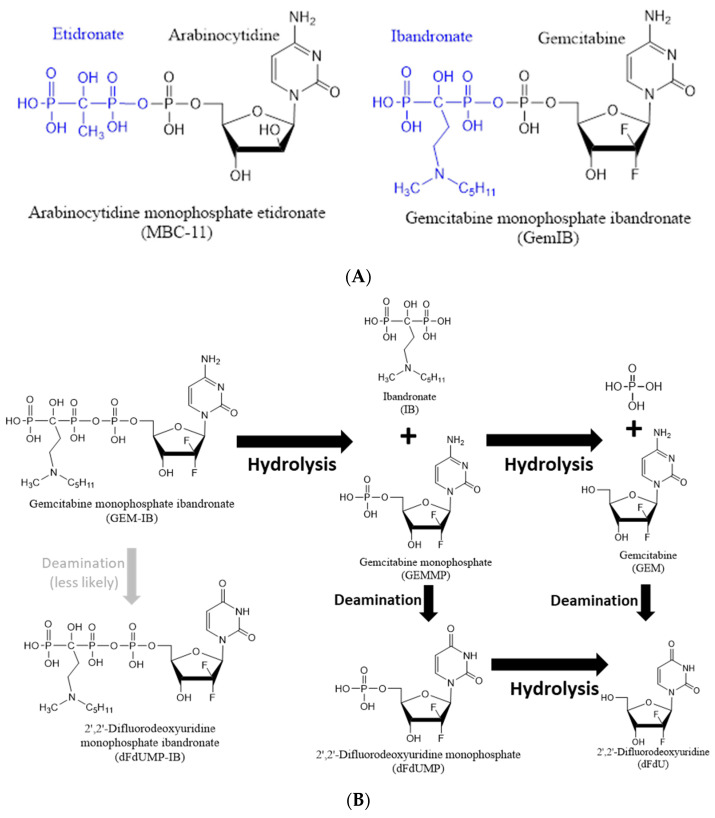
(**A**) Chemical structures of **MBC-11** [13] and **GEM-IB** [15]. Both drugs are the conjugates of a bisphosphonate bone-targeting component, linked together via phosphate group—**etidronate** and **arabinocytidine** (**MBC-11**) or **ibandronate** and **gemcitabine** (**GEM-IB**). (**B**) Chemical and metabolic breakdown of **GEM-IB**. **GEM-IB** is rapidly hydrolyzed to **gemcitabine-5′-phosphate** (**GEMMP**) and **ibandronate** (**IB**). **GEMMP** can be further hydrolyzed to **gemcitabine** (**GEM**) or enzymatically deaminated to **2′,2′-difluorodeoxyuridine-5′-phosphate** (**dFdUMP**). **2′,2′-Difluorodeoxyuridine** (**dFdU**) is formed either via deamination of **GEM** or hydrolysis of **dFdUMP**.

The exploitation of the bone-seeking properties of BPs for targeted delivery of cancer therapeutics [1,11,12,13,14] is a promising approach to target bone metastases. BPs and cytotoxic agents can be covalently linked, allowing the intact conjugate to leave the circulation and release both drugs in the bone microenvironment [1,13]. These conjugates could combine anti-resorptive and anti-tumor activities while localizing at the site of tumor cell-induced bone destruction. We have shown that **MBC-11** (see Figure 1), a first-in-class conjugate of the bone-targeting BP etidronate covalently linked to the antimetabolite **cytarabine** (**arabinocytidine** or **araC**), was well tolerated in humans and showed significant reductions in the metabolic activity of bone-associated cancer cells [13].

In the present study, we describe the development and validation of analytical platforms to quantify a novel drug in this class, a **gemcitabine–ibandronate conjugate** [15] (**GEM-IB**, see Figure 1A), and its metabolites (Figure 1B) and analyze its metabolism and pharmacokinetics (PK) in dogs and mice. **GEM** is one of the most widely used antineoplastic agents in clinical oncology, used to treat breast, ovarian, bladder, non-small lung, and pancreatic cancer [16,17]. As compared with other cytotoxic chemotherapy drugs, **GEM** is generally considered a tolerable compound for most cancer patients [17]. Treatment-related adverse events are usually clinically manageable and fatal treatment-related events have been reported in only a few (<1% to 4%) patients [17,18,19]. Side effects include hematological (e.g., neutropenia, anemia, thrombocytopenia, and thromboembolism) and non-hematological (e.g., vomiting, fatigue, and elevated levels of alanine aminotransferase) side effects [17]. The earliest studies of BP use for bone metastases involved trials of clodronate [20,21] and pamidronate [21,22] in the early 1990s. **Ibandronate** (**IB**) is an advanced BP medication used in the prevention and treatment of osteoporosis and metastasis-associated skeletal fractures [23]. **IB** is FDA-approved for the treatment and prevention of osteoporosis in post-menopausal women [24]. **GEM-IB** is a novel conjugate that combines the potent antineoplastic properties of **GEM** with the bone-targeting properties of **IB**. No studies describing the analysis, pharmacokinetics, and stability of this bisphosphonate conjugate have been conducted before.

In a manner analogous to **MBC-11**, **GEM-IB** is hydrolyzed under physiological conditions to form **gemcitabine-5′-phosphate** (**GEMMP**) and **IB**. While **IB** does not undergo further degradation, **GEMMP** is rapidly hydrolyzed to form **GEM** or is deaminated, resulting in **dFdUMP** (see Figure 1B). Hydrolysis of **dFdUMP** or deamination of **GEM** will result in the formation of the essentially inert metabolite **dFdU**. To investigate the absorption distribution and metabolism of **GEM-IB** after administration, it is important to monitor **GEM-IB** and the metabolites **GEMMP**, **IB**, **GEM**, **dFdUMP**, and **dFdU**. In the present study, we describe two novel analytical platforms for the analysis of **GEM-IB** and **IB** (Assay A) and **GEMMP**, **GEM**, **dFdUMP**, and **dFdU** (Assay B) in mouse and dog plasma to support preclinical studies. For the first time, we provide data from two preclinical studies, one in *mice* and one in *dogs*, to prove the feasibility of the approach and present the first set of PK data for this novel drug.

## 2. Results

**Figure 2:**
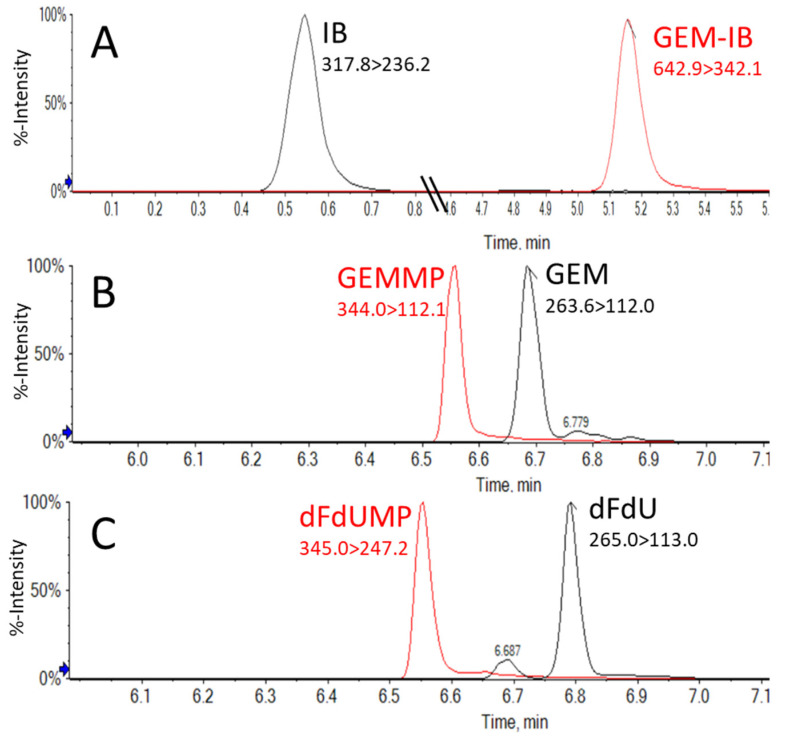
Assay A and Assay B’s chromatographic performance. A calibrator sample in mouse plasma is shown in all examples. (**A**) Representative extracted ion chromatograms of the highest calibrator sample for Assay A in mouse plasma for **IB** (4000 ng/mL) and **GEM-IB** (4000 ng/mL). Representative extracted ion chromatograms of the highest calibrator (1000 ng/mL) sample for Assay B in mouse plasma are shown in (**B**,**C**). Separation of **GEMMP** and **GEM** is shown in (**B**) and separation of **dFdU** and **dFdUMP** is shown in (**C**).

Various conditions were tested to achieve the acceptable chromatographic performance and separation of key components while maintaining optimal sensitivity, reproducibility, and minimal carry-over. Chromatographic performance for pyrimidine nucleosides and nucleotides (**GEM**, **dFdU**, **GEMMP**, and **dFdU-MP**) could be achieved under various common reversed-phase high-performance liquid chromatography (RP-HPLC) conditions. In contrast, achieving acceptable chromatography was challenging for **GEM-IB**. Normal-phase, reversed-phase (RP), and hydrophilic interaction liquid chromatography (HILIC) resulted in poor retention, retention reproducibility, and/or robustness for **IB** and **GEM-IB**. Ion-pair chromatography using various mass spectrometry-compatible ion-pair reagents in combination with RP-HPLC column materials showed an improved peak shape, but lacked reproducibility and sensitivity and showed a significant carry-over effect. This changed with the use of the Hypercarb column material, which consists of 100% porous graphitic carbon (PGC). Using this material and the conditions listed in Section 4 for Assay A, acceptable chromatographic performance was achieved (see Figure 2A). Unfortunately, the less polar metabolites (**GEM** and **dFdU**) did not show sufficient separation using PGC material under these conditions. Thus, a second chromatographic platform (Assay B) using simple RP-HPLC that proved more suitable for said metabolites was used to monitor pyrimidine nucleosides and nucleotides. Complete chromatographic separation of **GEM** and its monophosphate **GEMMP** as well as of **dFdU** from its monophosphate **dFdU-MP** was required due to in-source fragmentation. Figure 2B,C show representative extracted ion chromatograms for the analytes from extracted mouse plasma samples using RP-HPLC Assay B (please see Section 4).

### 2.1. Linearity, Lower Limit of Quantitation, and Selectivity

**Figure 3:**
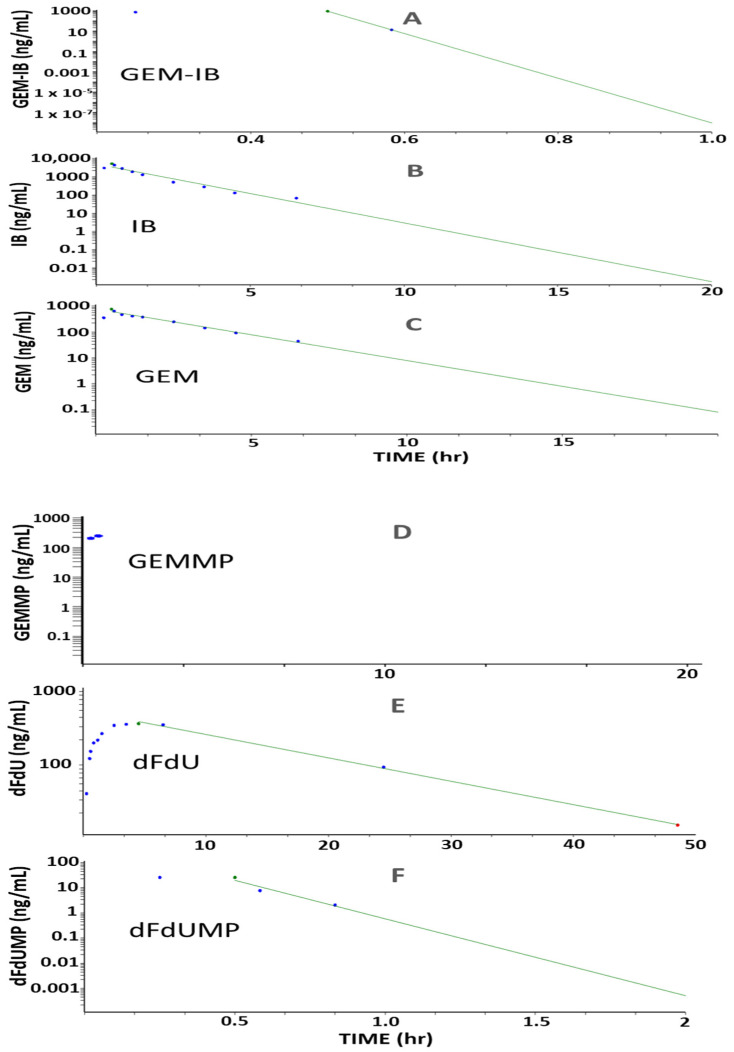
(Dog PK) Plot of PK profiles for **GEM-IB** and metabolites in dog plasma. For dogs (*n* = 3), **GEM-IB** (5 mg/kg) was infused i.v. for 30 min. Subfigures (**A**)–(**F**) are plots of the mean PK profiles for **GEM-IB**, **IB**, **GEM**, **GEMMP**, **dFdU**, and **dFdUMP**, respectively. Area under the concentration (AUC) versus time curves (green lines) are shown with extrapolation. Blue dots represent the mean concentrations observed. PK was calculated with Phoenix WinNonlin version 8.3, Certara, Princeton, NJ, USA.

The lower limit of quantitation (LLOQ) was defined as the lowest concentration for which the accuracies were within 20% of the nominal concentration for at least half of the samples and the imprecisions were less than 20%. The LLOQ also had a signal-to-noise (S/N) ratio greater than 8. The LLOQ was determined to be 5 ng/mL for **GEM**, **GEMMP**, and **dFdUMP**, while dFdU and **GEM-IB** had an LLOQ of 10 ng/mL and **IB** had an LLOQ of 40 ng/mL in dog and mouse EDTA plasma. The upper limit of quantitation (ULOQ) used in this study was 1000 ng/mL for all compounds, except for **IB**, which had a ULOQ of 4000 ng/mL. Representative extracted ion chromatograms of non-spiked blank mouse and dog plasma and spiked plasma and representative calibration curves for each compound are shown in Supplementary Figure S1a–f. The correlation coefficients for the calibration curves were consistently r = 0.99 and better. Figure 3 shows the chromatographic peaks for all compounds at a concentration of 2.5 ng/mL (below the lower limit), as well as the carry-over peaks for the solvent injection immediately following the injection with the highest concentration of sample (1000 ng/mL). The peak intensities of said injections indicate that the carry-over for this assay was less than 1% (See Supplementary Figure S1a–f).

### 2.2. Extraction Recovery and Matrix Effects

Matrix effects and extraction recovery was evaluated using the protocol described by Matuszewski et al. [25]. Six different individual lots of dog plasma and six different individual lots of mouse plasma were used for all recovery and matrix effect experiments. Plasma samples were enriched at three different levels within the working range of the assay and extracted (enriched before) and compared to a set of blank plasma samples that were extracted before the addition of analytes (enriched after). This was performed at three concentration levels corresponding to the high, mid, and low QC levels. The results of both sets of samples were compared and used to calculate the extraction recovery (Recovery in % = Signal area_enriched before_/Signal area_enriched after_ × 100). The resulting mean recovery and the coefficient of variation for each compound are shown in Table 1. The recovery of all compounds was calculated to be higher than 80%, with coefficients of variation from 4.4% to 25% (see Table 1). Finally, the six individual lots of dog plasma and the six individual lots of mouse plasma were also used to determine any effects that unique matrices had on the MS/MS signal (ion suppression/ion enhancement). To determine the matrix effect, the blank extracted samples that were enriched after extraction (enriched after) were compared to enriched buffer samples with the same organic/aqueous buffer combination as a sample extract (absolute matrix effect = Signal area_enriched after_/Signal area_buffer sample_ × 100, see Table 1). A value of >100% indicates ionization enhancement, and a value of <100% indicates ionization suppression. The relative matrix effect was calculated using the analyte/internal standard area ratio. This value shows if the internal standard can compensate for individual matrix effects (Table 1). The absolute matrix effect relative to the surrogate matrix showed the largest effect for **IB**, with 26.6% ± 2.4% (SD) and 31.2% ± 1.8% (SD) for mouse and dog plasma, respectively. For this analyte, the relative matrix effect showed a much higher value (suppression effect was mitigated by the internal standard), with 76.5% ± 3.2% (SD) and 102% ± 3.2% (SD) for mouse and dog plasma, respectively. The highest standard deviation (inter-individual variation) was observed for **GEM-IB**, with 137.7% ± 74.6% (SD) and 77.5% ± 34.3% (SD) for mouse and dog plasma, respectively. This variation was also compensated by the internal standard used, as shown by the relative matrix effect for **GEM-IB**, with 77.8% ± 11.7% (SD) and 77.1% ± 9.9% (SD) for mouse and dog plasma, respectively.

### 2.3. Matrix Interference

The six individual lots of both dog and mouse plasma were used to assess matrix interferences. For each lot, three levels of quality control samples that covered the working range of the assay were analyzed and the result was compared with the nominal concentration, for a total of *n* = 18 data points per compound. The calculated matrix interference of each compound is shown in Table 1. All compounds fulfilled the predefined acceptance criteria of a mean accuracy of 80–120% and mean imprecision of less than 20% for this experiment.

### 2.4. Accuracy and Imprecision

The accuracy and imprecision of this assay were tested by extracting calibrators and quality control samples on three separate days (runs). For each run, two sets of calibrators and six sets of quality control samples were extracted and analyzed. Five concentration levels were chosen for the analysis of **GEMMP**, **dFdU-MP**, and **GEM** (5, 10, 15, 200, and 800 ng/mL), while **dFdU** was tested at four concentration levels (10, 15, 200, and 800 ng/mL). dFdU was not tested at 5 ng/mL since it falls below the LLOQ. Both intra-batch and inter-batch accuracies and precisions were determined (see Supplementary Table S1). At least two-thirds of the calibration samples fell within 15% of the nominal concentration (or within 20% for the lowest concentration level). For quality control samples, at least half of the samples at each level and at least two-thirds of the samples overall fell within 15% of the expected concentration (or within 20% for the lowest concentration level). The coefficient of variation also fell within 15% (or 20% for the lowest concentration level) for all but two inter-batch quality control levels of **GEMMP**, which fell between 15% and 16%.

### 2.5. Stability

For stability testing, QC samples were prepared by spiking plasma with QC stock solutions. These stability samples were stored for the predetermined time periods before extraction (accelerated stability and freeze–thaw stability) or after extraction (autosampler stability) and were run with freshly prepared calibrator standards. For accelerated stability, samples were stored at 4 °C and at room temperature for 4 h and 24 h prior to sample extraction. Except for **GEM-IB**, all analytes were stable (±20% of nominal) for 24 h at room temperature for at least 24 h (see Supplementary Tables S2 and S3). Assay A compounds (**GEM-IB** and **IB**) were also tested at 1 h and 2 h exposed to 4 °C and at room temperature to evaluate for which duration **GEM-IB** is stable. While **IB** was stable under all conditions, **GEM-IB** was stable for up to 1 h on ice in mouse plasma with 89.2% ± 7.0% (mean ± SD) of the nominal concentration. After 2 h, **GEM-IB** had degraded to 64.2% ± 11.8% (mean ± SD). In contrast, **GEM-IB** in dog plasma was stable for up to 24 h on ice.

Freeze–thaw cycle stability was assessed by storing the stability samples overnight in the freezer (<−70 °C) and thawing them the next day on the bench. Once thawed, samples were placed back into the freezer. This was carried out for three freeze–thaw cycles for all compounds. Except for **GEM-IB** in mouse plasma, all study compounds were within ±20% of the nominal enriched concentration (see Supplementary Tables S5 and S6). Due to the instability of **GEM-IB**, one and two freeze–thaw cycles were tested for the Assay A compounds (**GEM-IB** and **IB**). While **IB** was stable under these conditions, **GEM-IB** showed instability after one freeze–thaw cycle (53.7% ± 6.9%).

The stability of extracted dog and mouse plasma samples in the autosampler at 4 °C was established for 24 and 48 h. Except for **GEM-IB**, in mouse and dog plasma extracts, all analytes were within ±20% of the nominal enriched concentration for up to 48 h in the autosampler. **GEM-IB** was stable for up to 24 h under these conditions.

To assess the stability of study samples, PK samples were re-extracted after storage at <−70 °C for 1.5 years (see Supplementary Table S4). Under these conditions, **GEM-IB** and IB showed an accuracy of 105.5% ± 40.4% (mean ± standard deviation) and 111.4% ± 38.0% for **GEM-IB** and 107.8% ± 2.5% and 113.7% ± 23.9% for **IB** in dog plasma and mouse plasma, respectively. Standard deviations were higher than the anticipated ±25% for **GEM-IB** but passed for IB. **GEM** was shown to be stable under these conditions, with accuracies of 90.4% ± 23.9% (mean ± standard deviation) and 94.2% ± 11.8% for dog and mouse plasma, respectively. Sample stability assessment for **GEMMP**, **dFdU**, and **dFdU-MP** failed the criteria in dog and mouse plasma at <−70 °C for 1.5 years (see Supplementary Table S4).

### 2.6. Proof-of-Concept PK Studies

**Figure 4:**
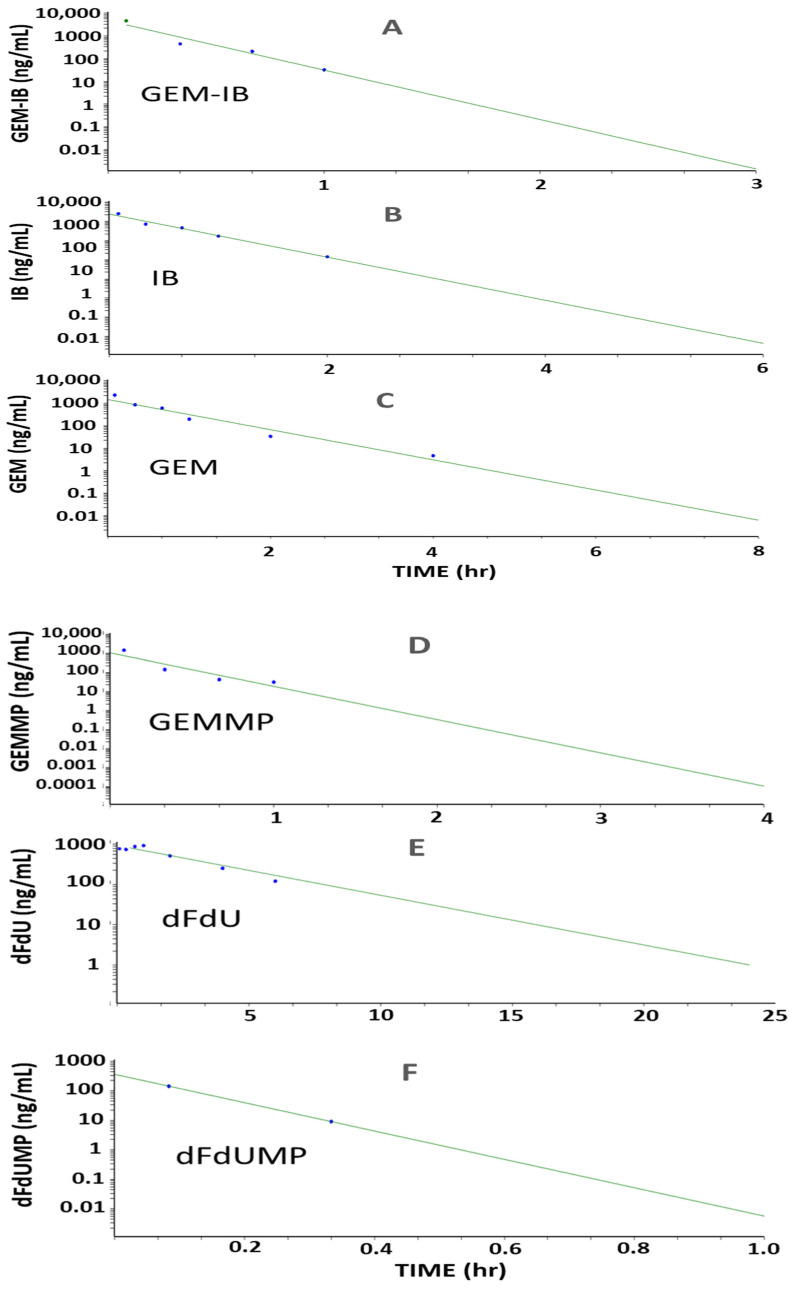
(Mouse PK) Plot of PK profiles for **GEM-IB** and metabolites in mouse plasma. *Mice* (*n* = 24) were injected i.p. with **GEM-I**B at 5 mg/kg. Subfigures (**A**)–(**F**) are plots of the mean PK profiles for **GEM-IB**, **IB**, **GEM**, **GEMMP**, **dFdU**, and **dFdUMP**, respectively. Area under the concentration (AUC) versus time curves (green lines) are shown with extrapolation. Blue dots represent the mean concentrations observed. PK was calculated using Phoenix WinNonlin (version 8.3, Certara, Princeton, NJ, USA).

**Figure 5:**
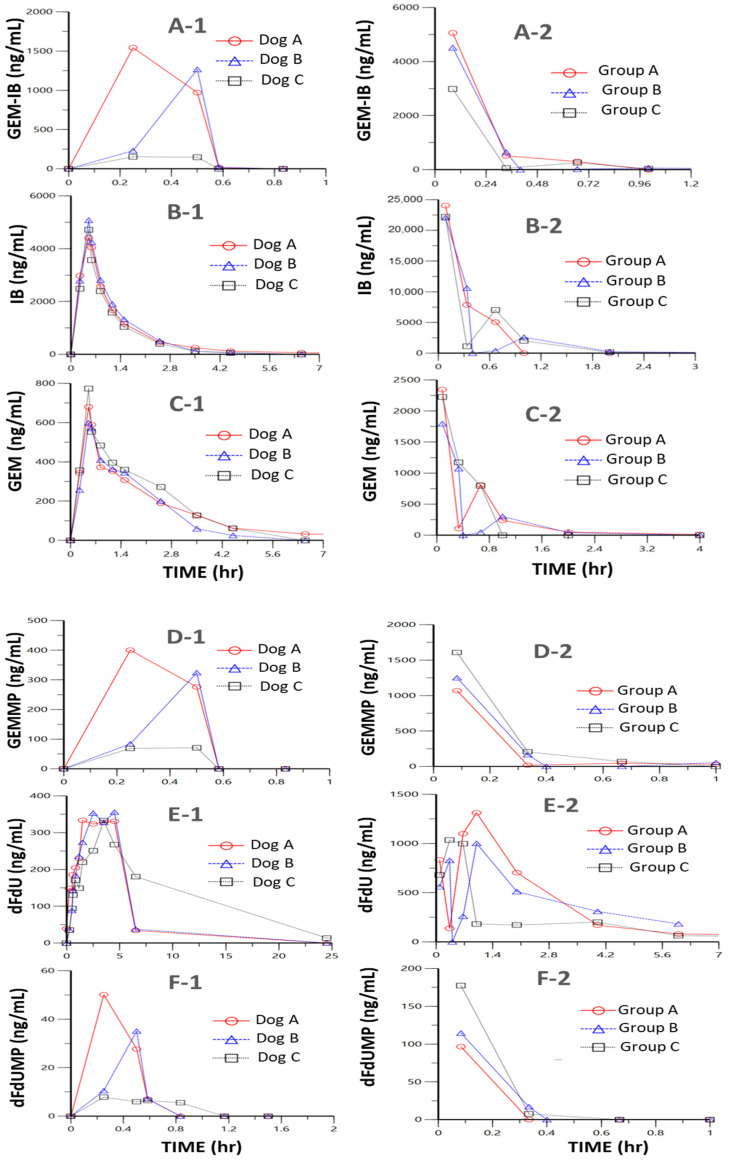
(Individual dog and mouse drug and metabolite levels.) Subfigures (**A-1**,**B-1**,**C-1**,**D-1**,**E-1**,**F-1**) show plots of the observed individual dog plasma drug and metabolite levels for **GEM-IB**, **IB**, **GEM**, **GEMMP**, **dFdU**, and **dFdUMP**, respectively. The mice were grouped into 3 groups (Group A, Group B, and Group C) and subfigures (**A-2**,**B-2**,**C-2**,**D-2**,**E-2**,**F-2**) show plots of the observed plasma drug and metabolite levels for **GEM-IB**, **IB**, **GEM**, **GEMMP**, **dFdU**, and **dFdUMP**, respectively.

Studies in mice (*n* = 24, eight time points, *n* = 3 per time point) and in dogs (*n* = 3, twelve time points) were performed to determine key PK parameters for **GEM-IB** and its metabolites in mice and dogs (Table 2 and Table 3 and Figure 3, Figure 4 and Figure 5). After i.p. injection in mice, the maximal concentrations (C_max_) were 4185, 22,777, 1309, 130, 2122, and 832 ng/mL for **GEM-IB, IB**, **GEMMP**, **dFdUMP**, **GEM**, and **dFdU**, respectively. All analytes showed a time of maximal concentration (t_max_) of 5 min (the first measured time point), with the exception of **dFdU**, which was formed with a delay (t_max_ = 1 h). The areas under the time concentration curve over the observation period (AUC_0-Ͳ_) in mouse plasma for **GEM-I**B, **IB**, **GEMMP**, **GEM**, **dFdU**, and **dFdU-MP** were 1278 h·ng/mL, 10,652 h·ng/mL, 405 h·ng/mL, 38 h·ng/mL, 1063 h·ng/mL, and 3389 h·ng/mL, respectively. In dog plasma, AUC_0-Ͳ_s of 295 h·ng/mL, 5725 h·ng/mL, 83 h·ng/mL, 11 h·ng/mL, 1625 h·ng/mL, and 6569 h·ng/mL were observed for **GEM-IB**, **IB**, **GEMMP**, **dFdU-MP**, **GEM**, and **dFdU**, respectively. The half-life of **GEM-IB** was determined to be 8 min after i.p. injection in mouse plasma and less than 1 min after the end of the infusion in dog plasma. Table 2 lists the key PK parameters determined in mice and Table 3 lists the key PK parameters in dogs. **GEMMP** could not be completely evaluated in dog plasma, since all values after the infusion period were below the LLOQ.

## 3. Discussion

**GEM-IB** is a novel **GEM-BP** conjugate that combines anti-resorptive and anti-tumor activities while localizing at the site of tumor cell-induced bone destruction. The exploitation of the bone-seeking properties of BPs (IB) for targeted delivery of chemotherapy (**GEM**) is a promising approach to target bone metastases. It was the aim of this study to develop and validate analytical platforms for the quantitative determination of **GEM-IB** plasma levels and consecutive analysis of the PK of **GEM-IB** and its metabolites in mouse and dog PK studies. The active metabolites **IB** and **GEM**, as well as another intermediate (**GEMMP**) and degradation products (**dFdU** and **dFdUMP**), were included.

### 3.1. Chromatographic Performance

Initially, various chromatographic conditions were tested for the separation of **GEM-IB**, **IB**, **GEM**, **GEMMP**, **dFdU**, and **dFdUMP**. While the separation of **GEM**, **GEMMP**, **dFdU**, and **dFdUMP** could be achieved using various different column materials, chromatographic analysis of **GEM-IB** and **IB** was challenging. For the chromatography of **GEM**, **GEMMP**, **dFdU**, and **dFdUMP** (Assay B), a Kinetex XB-C18 column (150 mm × 4.60 mm, 2.6 µm particle size, Phenomenex, Torrance, CA, USA) was chosen and 0.3% formic acid in HPLC-grade water (Buffer A) and acetonitrile (Buffer B) were used as mobile phases, resulting in good chromatographic separation of the study compounds, high sensitivity in positive ion mode, and a robust and reproducible chromatographic platform. As mentioned before, the development of a chromatographic assay for analytes **GEM-IB** and **IB** was more challenging. For Assay B, separation of the monophosphates from the nucleoside compounds (**GEM** from **GEMMP** and **dFdU** from **dFdUMP**) was critical because **GEMMP** can be converted to **GEM** and **dFdUMP** to **dFdU** during the ionization process in the electrospray ionization source. Ion-pairing has been used in numerous other studies involving the quantitation of nucleotides and oligonucleotides [26,27,28]. The use of ion-pairing allows chromatographic separation of polar compounds to be accomplished on a non-polar column, such as the C18 column used in this study. The amine moiety of **diethylamine** (**DEA**) binds to the polar groups of the analyte, in this case phosphate groups, while the hydrocarbon chains of **DEA** bind to the column media [29]. Initially, when ion-pair reagent was used for **IB** and **GEM-IB**, reasonable chromatographic performance could be achieved with minor tailing, but carry-over was excessive. Thus, several modifications were made to optimize the mobile phase buffer and different columns were tested. The use of a 10 mM **hexafluoroisopropanol** (**HFIP**) and 0.05% (*v*/*v*) **DEA** at pH 9.4 in combination with the novel Hypercarb (porous graphite) column stationary phase, which consists of 100% PGC, was the key to achieving good chromatographic separation with minimal carry-over. The use of PGC material was superior to the C8- and C18-based reversed-phase materials usually used in ion-pair chromatography. This could be due to the less lipophilic but more ionic nature of this relatively novel material. This platform resulted in the best separation and peak shape and minimized carry-over among the tested alternatives.

### 3.2. Assay Acceptance Criteria

As shown in Supplementary Table S1 and Table 1, the assay met the predefined acceptance criteria for intra- and inter-batch accuracy and imprecision. The specificity of the assay was evaluated by analysis of blank extracted plasma samples from six different individuals. The endogenous signal for the analytes was less than 20% of the LLOQ and there was no detectable signal for any of these compounds in the blank sample (see Supplementary Figure S1a–e). The extraction recovery was above 80% for all compounds (Table 1). Matrix interference also fell within ±15% of the nominal concentration for each compound (Table 1), indicating that the assays report accurate results independent of the individual matrix. Matrix interference testing demonstrated that the analytes could be analyzed accurately in individual matrices and that the internal standards compensated for matrix effects efficiently.

Matrix effects (ion suppression or enhancement) were studied because it is vital that all matrices behave similarly during extraction and analysis so that calibration samples that are created from bulk mouse and dog plasma will quantitatively represent the true concentrations of compounds in study samples. Possible matrix effects include ion suppression or enhancement, wherein components of the extracted plasma affect the ability of the compounds of interest to ionize within the source [25,27,30]. There was a pronounced absolute matrix effect for **IB** in mouse and dog plasma, with 26.6% ± 2.4% and 31.2% ± 1.8%, respectively. This effect can be expected for phosphorylated compounds, as described by our group for endogenous nucleotides [27] and by others [30]. However, standard deviations for the observed effect were low (2.4% and 1.8% for mouse and dog plasma, respectively), indicating that the effect is comparable/ similar amongst different matrices. In addition, the relative matrix effect, which normalized the area of the analytes by the area of the internal standards, showed improved values of 76.5% ± 3.2% and 102.0% ± 3.2% for **IB** in mouse and dog plasma, respectively. This indicated that the internal standards were able to compensate for the matrix effect. The matrix interference test ultimately determines if the measured matrix effects have an impact on the accurate determination of the analytes in individual matrices. All compounds fulfilled the predefined acceptance criteria for this test. Therefore, the observed matrix effects are comparable in all mouse and dog plasma samples and have no impact on the accurate determination of **GEM-IB**, **IB**, **GEM**, **GEMMP**, **dFdU**, and **dFdUMP** in mouse and dog plasma matrices, as demonstrated by the acceptable intra- and inter-batch accuracies and imprecisions.

Intra- and inter-batch accuracies and imprecision were determined over the duration of three days for mouse and dog plasma. The predefined acceptance criteria included 85% to 115% for accuracy (80–120% at the LLOQ) and a coefficient of variation (CV) of less than 15% for imprecision. These were applied to the mean accuracy and imprecision (*n* = 6 per level and day). In addition, two-thirds of all validation quality control samples overall and 50% of samples at a given level had to fulfill the accuracy acceptance criteria. All analytes met these predefined acceptance criteria for the three validation days.

### 3.3. Stability Assessments

An accelerated stability evaluation for up to 24 h on ice or at ambient temperature revealed that all analytes except for **GEM-IB** were stable under the tested conditions. **GEM-IB** was designed to be hydrolyzed under physiological conditions (see Figure 1B), with the **IB** moiety guiding and anchoring **GEM-IB** to the bone and hydrolysis enabling the release of the potent antineoplastic **GEM**. This drug design feature required extra attention to be paid to **GEM-IB** stability throughout the development of analytical methods. The number of freeze–thaw cycles that samples can be exposed to before the analytes start degrading and whether there is an effect of freeze–thaw cycles was investigated. Except for **GEM-IB**, all compounds were stable after exposure to three freeze–thaw cycles. Repeated experiments showed that **GEM-IB** was unstable after one freeze–thaw cycle. This could be due to the experimental design, which included thawing samples on the benchtop and then placing these back into the freezer. The instability of **GEM-IB** on the benchtop at ambient temperature was due to hydrolysis. In addition, **GEM-IB** showed instability after one freeze–thaw cycle (54%). However, extracts of **GEM-IB** remain stable for extended durations. Therefore, it is recommended to divide **GEM-IB** mouse plasma prior to freezing into 100 μL aliquots and add the protein precipitation solution to the frozen aliquot prior to thawing at 4 °C on ice. This will eliminate hydrolysis when thawing in plasma. Extracted samples were stable in the autosampler at 4 °C for 24 h (**GEM-IB**) and even up to 48 h (all other analytes). Reanalysis of PK study samples after 1.5 years (**GEM-IB** and **IB**) and 2.5 years (**GEM**, **GEMMP**, **dFdU**, and **dFdUMP**) revealed that only **GEM** and **IB** could be considered stable for this duration. Although mean accuracies for **GEM-IB** were within the acceptable range of 80–120% accuracy, standard deviations were high (40.4%), indicating variability across individual samples during long-term storage. Thus, extended storage (1.5 years) results in inaccuracy for **GEM-IB** in individual samples and is not recommended. The data for **GEM** are in alignment with the literature, since others have shown that **GEM** is stable in human plasma for a duration of up to 30 days at −70 °C [31] and also that **GEM** is stable in mouse plasma for a duration of up to 42 days [32]. It has also been shown that **IB** is stable in human plasma for up to 4 months at −20 °C [33]. However, **dFdU** has been shown to be stable for up to 42 days in enriched mouse plasma [32], which was not the case for the re-extracted samples that were stored at <−70 °C in the present study. Comparable stability data for **GEM-IB**, **GEMMP**, and **dFdU** are currently not available in the literature.

### 3.4. PK Studies

**GEM-IB** [15] was designed to improve upon the therapeutic benefits observed in clinical trials [12,13] of **MBC-11**. While direct measurement in the bone matrix was not made of either **MBC-11** nor released cytarabine, bone localized effects proved 2–20-fold greater compared to free cytarabine [13]. This observation suggests that the conjugation of etidronate to cytarabine not only drives bone localization, but also inhibits the inactivating deamination of cytarabine. **MBC-11** plasma PK also indicated that hydrolysis occurred on the minute time scale while in circulation. We hypothesize that vascular endonucleotidases catalyze the hydrolysis. The larger steric bulk of **GEM-IB**’s **IB** moiety compared to **MBC-11’s** etidronate moiety is thought to reduce the rapid hydrolysis and further deamination of the **GEM** moiety, all with the potential to increase the dose fraction delivered to the bone. The PK studies presented here determine key plasma PK parameters of **GEM-IB** and metabolites after the administration of 5 mg/kg of the study drug (**GEM-IB**) via i.p. and 30 min i.v. injection in mice and dogs, respectively. The minute-long half-lives for **GEM-IB** (8 min after i.p. injection in mouse plasma and less than 1 min after the end of the infusion in dog plasma) indicated that **GEM-IB** was rapidly distributed or hydrolyzed, similar to what was observed with **MBC-11**. The major metabolite was **IB**, the hydrolysis product of **GEM-IB**. **IB** had estimated half-lives of 16 min in mice and 55 min in dogs (Table 2). Maximal plasma concentrations (C_max_) differed between dogs and mice and were generally higher in mice. However, this can also be explained by the route of administration (i.p. vs. i.v.). Surprisingly, relatively high AUC_0-Ͳ_s of **IB** were observed after administration in mice (mean: 10,652 h·ng/mL), while the sum of all **GEM**-related metabolites added up to 4895 h·ng/mL. This was not the case for dogs with AUC_0-Ͳ_s for **IB** of 5725 h·ng/mL and 8289 h·ng/mL as the sum of all **GEM**-related metabolites. This might be indicative of differences in the metabolism of **GEM-IB** between these species. Mice may be able to produce **GEM** metabolites that were not captured by this assay. Moreover, the observed differences in the pharmacokinetic parameters are likely at least in part explained by the different routes of administration (i.p. vs. i.v.), resulting in different distribution and hydrolysis rates. The plasma PK behaviors of **GEM**, **dFdU**, and **IB** after the hydrolysis of **GEM-IB** are consistent with the well-known PK values established by others when **GEM** or **IB** are administered as free drugs [34,35]. The development and use of the presented plasma PK methods provides the foundation for future work, asking the critical questions of the dose fraction that is bone-localized and the bone-localized levels of **GEM** vs the inactivated deaminated **dFdU** that drive efficacy.

## 4. Materials and Methods

To analyze **GEM-IB** and its five metabolites, two analytical assays were developed. Assay A captures the polyphosphate-containing compounds **GEM-IB** and **IB**. Assay B was designed to analyze the nucleotide analog metabolites including **GEMMP**, **dFdUMP**, **GEM**, and **dFdU**.

### 4.1. Materials

**Gemcitabine hydrochloride** and **2′,2′-difluoro-2′-deoxyuridine** were purchased from Thermo Fisher Scientific (Waltham, MA, USA). **GEM-IB** was synthesized according to a published procedure [15] and **2′,2′-Difluoro-2′-deoxyuridine-monophosphate** was prepared by standard deamination of **GEMMP**. **GEMMP** and the internal standards, **gemcitabine-^13^C,^15^N_2_** (**GEM-^13^C**) and **2′,2′-difluoro-2′-deoxyuridine-^13^C,^15^N_2_** (**dFdU-^13^C**), were purchased from Toronto Research Chemicals (North York, ON, Canada). Ibandronate sodium salt was purchased from Sigma Aldrich (St. Louis, MO, USA). **Adenosine-^13^C_10_,^15^N_5_ 5′-triphosphate** (**^13^C_10_-^15^N_5_-ATP**) was purchased from Sigma Aldrich (St. Louis, MO, USA). K_2_EDTA canine Beagle plasma and mouse plasma used for the assay validation were purchased from Innovative Research (Novi, MI, USA). HPLC-grade **water**, **methanol**, **acetonitrile**, **formic acid**, and **dibutylamine** (**DBA**) were purchased from Thermo Fisher Scientific (Waltham, MA, USA).

### 4.2. Stock Solutions

Individual stocks of all compounds (**GEM-IB**, **GEMMP**, **IB**, **GEM**, **dFdU**, and **dFdUMP**) were prepared by weighing out solid material and dissolving it in HPLC-grade **water** to achieve a final concentration of 1 mg/mL.

### 4.3. Calibrators and Quality Controls

Calibration and quality control samples were prepared by spiking 180 µL of plasma with 20 µL of the corresponding calibrator or quality control stock, as listed above.

For **Assay A** (analysis of **GEM-IB** and **IB**), combined stock solutions with 10 µg/mL of **GEM-IB** and 40 µg/mL **IB** were prepared. These were used to prepare calibrator stock solutions used for the enrichment of mouse or dog plasma. Calibrator standards were prepared at concentrations of 2.5, 5, 10, 25, 50, 100, 250, 500, and 1000 ng/mL for **GEM-IB** and 10, 20, 40, 100, 200, 400, 1000, 2000, and 4000 ng/mL for **IB**. Quality control samples (QC_LLOQ_, QC_low-1_, QCl_ow-2_, QC_mid_, and QC_high_) were prepared at 5, 10, 30, 200, and 800 ng/mL for **GEM-IB** and 20, 40, 120, 800, and 3200 ng/mL for **IB**.

For **Assay B** (analysis of **GEMMP**, **GEM**, **dFdUMP**, and **dFdU**), combined stock solutions with all four compounds were prepared at a concentration of 10 µg/mL from the individual 1 mg/mL stocks. These were used to prepare the calibrator stock solutions used for the enrichment of mouse/dog plasma. Calibrator standards were prepared at concentrations of 2.5, 5, 10, 25, 50, 100, 250, 500, and 1000 ng/mL. Quality control samples (QC_LLOQ_, QC_low-1_, QCl_ow-2_, QC_mid_, and QC_high_) were prepared at concentrations of 5, 10, 15, 200, and 800 ng/mL. A protein precipitation solution was prepared by adding 250 ng/mL of each internal standard (**GEM-^13^C** and **dFdU-^13^C**) to 20 mM DBA in methanol. All stocks were stored at −20 °C.

### 4.4. Sample Extraction

**Assay A** (**GEM-IB** and **IB**): The extraction of calibration standards, quality control samples, and PK study samples was identical. Plasma, quality control stocks, and calibrator stocks were removed from the storage freezer and kept at room temperature until fully thawed. Aliquots of 100 μL of standards/quality control or blank sample were transferred into a 1.5 mL low-binding polypropylene vial with a conical bottom and snap-on lid. An amount of 10 µL of the of the internal standard solution (15 and 10 µg/mL of **IB-D_3_** and **ATP-IS**, respectively) was added and mixed. For protein precipitation, 50 μL of methanol was added and then samples were vortexed for 10 min. This was followed by centrifugation at 25,000× *g* for ten minutes at 4 °C (Thermo Scientific MR 23i Centrifuge, Thermo Scientific, Waltham, MA, USA). To remove remaining protein, the supernatant was transferred to 10,000 molecular weight cut off (MWCO) centrifugal filters (800 µL volume, PES 10,000 with 2.0 mL receiver tubes, Analytical Sales and Services, Flanders, NJ, USA) and centrifuged again at 25,000× *g* for ten minutes at 4 °C. The filtrate was transferred into HPLC vials and placed into the HPLC autosampler maintained at 4 °C.

**Assay B** (**GEMMP**, **GEM dFdUMP** and **dFdU**): The extraction of calibration standards and quality control samples was identical to that of study samples. An amount of 200 µL of plasma sample was added to a 1.5 mL snap-top Eppendorf tube with 100 µL of protein precipitation solution. The samples were then vortexed for five minutes followed by centrifugation at 25,000× *g* for ten minutes at 4 °C. Hereafter, supernatants were transferred to 10K Amicon centrifugal filters (Merck-Millipore, Burlington, MA, USA) and centrifuged at the same settings for 60 min. The filtrates were then transferred into HPLC vials for analysis.

### 4.5. HPLC-MS/MS Analysis

**Assay A** (**GEM-IB** and **IB**): Quantification of the analytes was carried out using high-performance liquid chromatography–tandem mass spectrometry (HPLC-MS/MS). Chromatography was performed using an Agilent 1260 Infinity II bio-inert HPLC system (Agilent Technologies, Santa Clara, CA, USA) on a Hypercarb Porous Graphitic Carbon HPLC column (3 µm, 2.1 × 50 mm, Thermo Scientific, Waltham, MA, USA). The HPLC system consisted of an Agilent 1260 Infinity II bio-inert quaternary pump, an Agilent 1260 Infinity II bio-inert column oven, and an Agilent 1260 Infinity II bio-inert autosampler (Agilent Technologies, Santa Clara, CA, USA). An amount of 10 µL of the sample extracts was injected. The column was maintained at 22 °C. HPLC-MS-grade water supplemented with 10 mM hexafluoroisopropanol (HFIP) and 0.05% (*v*/*v*) diethyl amine (DEA) at pH 9.4 was used for the aqueous mobile phase (Buffer A) and HPLC-grade acetonitrile was used for the organic mobile phase (Buffer B). The flow rate was set to 400 µL/min throughout the assay. The initial settings were 98% Buffer A and 2% organic Buffer B for the first two minutes. During the following 0.7 min, the organic Buffer B was increased to 30%. At minute 3.5, the gradient reached 98% Buffer B, which was kept for 0.5 min, and at minute 4.2 the column was re-equilibrated to starting conditions for 2.8 min until the next injection. The HPLC system was connected to a Sciex a 5500+ triple quadrupole mass spectrometer (SCIEX, Concord, ON, Canada) via a turbo electrospray ionization source (SCIEX) operated in the negative electrospray ionization (ESI) mode. Data were acquired in negative multiple reaction monitoring (MRM) mode and all compounds were detected as [M–H ]^−^. The ion transitions used during the multiple reaction monitoring mode were *m*/*z* = 318→236, 321→239, 643→342, and 521→79 for **IB**, **IB-D_3_**, **GEM-IB**, and **adenosine-^13^C_10_,^15^N_5_ 5′-triphosphate** (**^13^C_10_-^15^N_5_-ATP**), respectively. The dwell time for analysis was set to 50 ms. The curtain gas was kept at 20 psi and the ion source gas at 55 psi. The ion spray voltage was −4500 volts, and the temperature of the ion source was set to 400 °C. Compounds were quantified using the analyte/internal standard area ratios based on calibration curves that were constructed with 1/x weighting and a linear regression fit. Quantification was carried out using the Sciex Analyst 1.7.1 Software (SCIEX, Foster City, CA, USA).

**Assay B** (**GEMMP**, **GEM**, **dFdUMP** and **dFdU**): Quantification of the analytes was carried out using HPLC-MS/MS. Chromatography was performed using an Agilent 1100 series HPLC system (Agilent Technologies, Santa Clara, CA, USA) equipped with a Kinetex XB-C18 column (150 mm × 4.60 mm, 2.6 µm particle size, Phenomenex, Torrance, CA, USA). The HPLC system consisted of an Agilent 1100 series binary pump, an Agilent 1100 degasser, an Agilent 1260 column oven, and a LEAP PAL autosampler (CTC Analytics/Archer Science, Lake Elmo, MN, USA) that was set to inject 8 µL of sample. The column was maintained at 30 °C. HPLC-grade water with 0.3% (*v*/*v*) formic acid was used for the aqueous mobile phase (Buffer A) and HPLC-grade acetonitrile (Buffer B) was used for the organic mobile phase. The flow rate was set to 600 µL/min throughout the assay. The initial settings were 99% Buffer A and 1% organic Buffer B for the first minute. During the following 1.2 min, the organic Buffer B was increased to 10% and within the following 1.3 min to 40%. At minute 4.5, the gradient reached 85% Buffer B and within the following 0.2 min the gradient was ramped up to 99% Buffer B. The 99% Buffer B was kept for 1.8 min and at minute 6.6, the column was re-equilibrated at the starting conditions for 0.9 min until the next injection. The HPLC system was connected to a Sciex a 6500 QTRAP mass spectrometer (SCIEX, Concord, ON, Canada) via a turbo electrospray ionization source (SCIEX) operated in positive electrospray ionization (ESI) mode. The MS/MS was run in the positive multiple reaction monitoring mode and all compounds were detected as [M+H]^+^. The ion transitions used during the multiple reaction monitoring mode were *m/z =* 344→112, 345→247, 264→112, 265→113, 267→115 and 268→116 for **GEMMP**, **dFdUMP**, **GEM**, **dFdU**, **^13^C_3_-GEM**, and **^13^C_3_-dFdU**, respectively. The dwell time was set to 20 milliseconds. The curtain gas was kept at 20 psi and the ion source gas at 40 psi. The ion spray voltage was 5500 volts, and the temperature of the ion source was set to 550 °C. The compounds were quantified using the analyte/internal standard area ratios based on calibration curves that were constructed with 1/x weighting and a linear regression fit. Quantification was carried out using the Sciex Multiquant OS Software version 1.7 or higher (SCIEX, Foster City, CA, USA).

### 4.6. Assay Validation

Method development and validation was conducted following applicable FDA guidelines for bioanalytical assays [36], as considered fit for purpose. **Specificity.** Endogenous interferences were excluded by analysis of blank plasma from 6 different individual lots of plasma. The lower limit of quantification (LLOQ) was determined as the lowest concentration consistently achieving an accuracy better than ±20% of the nominal concentration, with imprecision ≤20%. **Predefined acceptance criteria.** The performance of the assay was considered acceptable if intra- and inter-day imprecision (coefficient of variance, %CV) at each concentration was ≤15%, except at the LLOQ (≤20%). Intra- and inter-day accuracy had to be ±15% of the nominal value, except at the LLOQ (±20%). Calibration curves had to have a correlation coefficient (r) of 0.99 or better. **Analytical recovery and imprecision.** Intra- and inter-day analytical accuracy and imprecision were tested at least at four concentration levels depending on the LLOQ for each compound. Intra-day imprecision and accuracy were determined with *n* = 6 per QC level. Inter-day imprecision and accuracy were measured over 3 days, with six replicates for each QC concentration (*n* = 18). **Extraction efficiency and matrix effect.** Extraction efficiency for **GEM-IB** and metabolites was established by comparing the signals of the analytes after the extraction of QCs (*n* = 6/concentrations), with the signals of the extracted blank matrix spiked with the respective concentrations of analytes after the extraction procedure [25]. The matrix effect was determined by comparing the analyte LC-MS/MS signal after spiking of the analyte into the extracted blank matrix (matrix from 6 different individual lots, analyte concentrations as for the QC samples) with the HPLC-MS/MS signal of the same amount of analyte in neat solution [25]. **Carry-over.** Carry-over was assessed by injecting blank buffer samples after analysis of the highest calibrators. Carry-over was considered insignificant if there was no signal at the relevant retention times exceeding 20% of the analyte signals at the LLOQ. **Stability.** QC samples were freshly prepared. One set was extracted and was analyzed immediately to determine baseline signals. Analyte stability in plasma was evaluated at two QC concentration levels (*n* = 3/concentration level) under different conditions: 1, 2, and 24 h at room temperature and at 4 °C. In addition, stability after extraction was determined after 24 h and 48 h at 4 °C in the autosampler and for up to 3 freeze (−70 °C)–thaw (ambient) cycles. Stability was assessed by comparing analyte concentrations with baseline (t_0_) concentrations. Stability was assumed when the results were within ±20% of the baseline concentrations. Long-term stability estimates were performed by re-extraction of samples used during the PK study (*n* = 6). Samples were initially analyzed for the PK assessment and were stored for 1.5 years (**GEM-IB** and **IB**) and 2.5 years (**GEM**, **GEMMP**, **dFdU**, and **dFdU-MP**) at <−70 °C prior to reanalysis. Stability was assessed by comparing analyte concentrations with the initial assessments. Stability was acceptable when the results were within ±20% of the baseline concentrations and the percent standard deviation (%-SD) was below 25%.

### 4.7. Proof-of-Concept Studies

**Dog PK study.** After completion of the validation, the assays were used to analyze plasma samples that were collected for a study investigating the PK of **GEM-IB** in dogs. For this proof-of-concept study, healthy beagle dogs (*n* = 3) received 5 mg/kg **GEM-IB** i.v. over the duration of 30 min. Plasma samples for PK analysis were collected before and during the infusion period (30 min) at 0, 15, and 30 min and after infusion ended at minutes 5, 20, 40, 60, 120, 180, 240, and 360. Blood samples were collected in K_2_EDTA containers with tetrahydrouridine 0.25 mg/mL to prevent deamination and plasma was separated. Samples were frozen and stored below −70 °C until HPLC-MS/MS analysis. **GEM-IB** and **IB** were assessed using the procedure described for Assay A and **GEMMP**, **dFdUMP**, **GEM**, and **dFdU** were analyzed using the protocol described under Assay B. **Mouse PK study.** Another study was performed as a proof-of-concept study to investigate the pharmacokinetics of **GEM-IB** in *mice*. Twenty-four mice were randomly assigned to three sequences. Each sequence consisted of eight mice and the plasma of each mouse was collected at one of the eight consecutive time points. Mice were injected with 5 mg/kg **GEM-IB** in saline i.p. Eight time points (with *n* = 3 mice per time point) were investigated and 0.25 mg/mL K_2_EDTA-tetrahydrouridine to prevent the deamination of blood samples was drawn via cardiac puncture at 5, 20, 40, 60, 120, 240, and 360 min. Plasma was separated, and samples were frozen and stored below −70 °C until HPLC-MS/MS analysis. **GEM-IB** and **IB** were assessed using the procedure described for Assay A and **GEMMP**, **dFdUMP**, **GEM**, and **dFdU** were analyzed using the protocol described under Assay B. The experimental protocols and animal care were in accordance with the Guide for the Care and Use of Laboratory Animals (Institute of Laboratory Animal Resources, 1996) and were approved by the Institutional Animal Care and Use Committee of Colorado State University (Ft. Collins, CO, USA). The animals were housed in an AAALAC-accredited and USDA-registered facility and the studies were carried out under the supervision of a board-certified veterinarian.

### 4.8. Statistics

All values are expressed as mean ± standard deviation (SD). Quantitative data were compared with analysis of variance (ANOVA), followed by Tukey’s post hoc test for multiple comparisons, using SPSS statistics version 28.0.1.0 (IBM, Armonk, NY, USA). A value of *p* < 0.05 was considered statistically significant. Intra- and inter-batch accuracies and precisions were calculated using Watson LIMS software version 7.6 (Thermo Fisher Scientific, Philadelphia, PA, USA). Non-compartmental PK parameters were calculated using Phoenix WinNonlin version 8.3 (Certara, Princeton, NJ, USA) or by the integrated PK module of Thermo Fisher Scientific Watson LIMS software (version 7.6, SP1).

## 5. Conclusions

Overall, the present assays for the quantification of **GEM-IB**, **IB, GEMMP**, **dFdUMP**, **GEM**, and **dFdU** in mouse and canine plasma met the pre-defined acceptance criteria for sensitivity, selectivity, specificity, precision, and accuracy. Extraction recovery and matrix interference met the acceptance criteria, and matrix effects could be compensated for by the internal standards. The assay was successfully applied to the analysis of study samples from two proof-of-concept PK studies in mice and dogs. Key PK parameters for **GEM-IB** and its metabolites were determined. The rapid distribution and metabolism of **GEM-IB** will require further investigation in follow-up studies. In said studies, tissue and bone material needs to be collected and analyzed to further investigate the bone accumulation of **GEM-IB** and its metabolites.

## Figures and Tables

**Table 1:** Recovery, matrix effects, and matrix interference in mouse and dog EDTA plasma.

	Recovery		Absolute Matrix Effect		Relative Matrix Effect		Matrix Interference	
	Mouse	Dog	Mouse	Dog	Mouse	Dog	Mouse	Dog
	%-Recovery	%-Recovery	%-ME	%-ME	%-ME	%-ME	%-Accuracy	%-Accuracy
GEM	103.3 ± 10.6	102.8 ± 17.4	73 ± 17.6	66.9 ± 12.3	76.4 ± 7	56.1 ± 5.4	97.7 ± 11.7	102.8 ± 17.4
dFdU	101.1 ± 14.2	96.6 ± 19.5	79.4 ± 10.3	81 ± 8.1	80.6 ± 12.6	55.6 ± 7.3	99.3 ± 14.6	96.6 ± 19.5
GEMMP	97 ± 13.6	107 ± 17.6	72.5 ± 12	65.2 ± 12.7	76.7 ± 11.4	55 ± 6.8	101.3 ± 13.9	107 ± 17.6
dFdUMP	87.2 ± 15.1	97.2 ± 16.5	62.8 ± 11.8	55.7 ± 10.6	63.4 ± 7.3	38.1 ± 5.3	91.3 ± 12.4	97.2 ± 16.5
IB	103.1 ± 11.1	99 ± 4.4	26.6 ± 2.4	31.2 ± 1.8	76.5 ± 3.2	102 ± 3.2	102 ± 2.8	102.7 ± 2
GEM-IB	80.7 ± 25	94.4 ± 6.3	137.7 ± 74.6	77.5 ± 34.3	77.8 ± 11.7	77.1 ± 9.9	103.7 ± 9.1	101.1 ± 13.4

Data are presented as means ± standard deviation.

**Table 2:** Key PK parameters after **GEM-IB** administration (i.p.) in *mice* (*n* = 3 *mice* per time point, 8 timepoints, 24 mice total). The non-compartmental PK analysis was carried out based on the mean concentrations for each time point. In mice, each time point was collected from a different animal.

Parameter	Units	GEM-IB	IB	GEM	GEMMP	dFdU	dFdUMP
C_max_	ng/mL	4185	22,777	2122	1309	832	130
T_max_	h	0.083	0.083	0.083	0.083	1.0	0.083
AUC	h·ng/mL	1278	10,652	1063	405	3389	38
R-Squared		0.946	0.987	0.954	0.848	0.991	1.000
No of points		4	5	6	4	5	2
Rate Constant	1/h	5.0	2.6	1.5	4.0	0.4	11.1
T_1/2_	h	0.138	0.268	0.448	0.172	1.798	0.063
Route		IV Bolus	IV Infusion	IV Infusion	IV Infusion	IV Infusion	IV Infusion
CL	mL/h/kg	3939	509	4714	12,647	1861	135,477
Vd_ss_	mL/kg	469	198	2063	1573	4869	8994

Abbreviations: C_max_, maximum (peak) plasma concentration; T_max_, time to peak drug concentration; AUC, area under the curve, representing the total drug exposure across time; T_1/2_, half-life, which is the time it takes for half the drug to be eliminated; CL, systemic clearance; Vd_ss_, volume of distribution.

**Table 3:** Key PK parameters after **GEM-IB** (30 min i.v.) administration in dogs (means, *n* = 3).

Parameter	Units	GEM-IB	IB	GEM	GEMMP	dFdU	dFdUMP
C_max_	ng/mL	795	4737	684	224	330	23
T_max_	h	0.5	0.5	0.5	0.5	4.5	0.5
AUC	h·ng/mL	295	5725	1625	83	6569	11
R-Squared		1.000	0.944	0.985	ND	0.991	0.873
No points		2	9	9	0	6	3
Rate Constant	1/h	50.6	0.7	0.5	ND	0.1	7.0
T_1/2_	h	0.014	0.932	1.511	ND	9.845	0.099
Route		IV Infusion	IV Infusion	IV Infusion	IV Infusion	IV Infusion	IV Infusion
Infusion Time	h	0.5	0.5	0.5	0.5	0.5	0.5
CL	mL/h/kg	17,021	956	3705	ND	738	448,855
Vd_ss_	mL/kg	1934	1037	7852	ND	9533	66,286

Abbreviations: C_max_, maximum (peak) plasma concentration; T_max_, time to peak drug concentration; AUC, area under the curve, representing the total drug exposure across time; T_1/2_, half-life, which is the time it takes for half the drug to be eliminated; CL, systemic clearance; Vd_ss_, volume of distribution. ND—not detected.

## Data Availability

All data from this study are available at the archives and secure servers of iC42 Clinical Research and Development, Department of Anesthesiology, University of Colorado, Aurora, Colorado, USA. Data can be made available upon reasonable request.

## Supplementary Materials

The following supporting information can be downloaded at https://www.mdpi.com/article/10.3390/molecules30020354/s1: Figure S1: Representative calibration curves and representative extracted ion chromatograms. Figures S1.a and S1.b show calibration curves and representative extracted ion chromatograms for **IB** and **GEM-IB** in mouse plasma (S1.a) and dog plasma (S1.b). A and B show calibration curves for **IB** and **GEM-IB**, C and D show extracted ion chromatograms of blank extracted mouse samples for **IB** and **GEM-IB** (S1.a C and D) and dog plasma for **IB** and **GEM-IB** (S1.b C and D). The row below (E and F) shows calibrator samples at the lower limit of quantitation 40 ng/mL and 10 ng/mL for **IB** and **GEM-IB** respectively. H and G show representative extracted ion chromatograms of **IB** and **GEM-IB** in study samples after infusion with **GEM-IB**. Similarly, Figure S1.c A-B and Figure S1.d A-B show representative calibration curves for **GEM** (A) and **GEMMP** (B). The following 3 rows show blank extracted matrix (C and D), the lower limit of quantitation samples (E and F) and study samples for **GEM** and **GEMMP** (G and H) in mouse (S1.c C to H) and dog plasma (S1.d C to H). Finally, the **dFdU** and **dFdUMP** in mouse and dog plasma is displayed in Figure S1.e and S1.f, respectively. Figure S1.e A-B and Figure S1.f A-B show representative calibration curves for **dFdU** (A) and **dFdUMP** (B) and the following rows show blank extracted matrix (C and D), the lower limit of quantitation samples (E and F) and study samples for dFdU and **dFdUMP** (G and H) in mouse (S1.e C to H) and dog plasma (S1.f C to H); Table S1: Intra- and inter-run accuracy and imprecision; Table S2: Accelerated stability in mouse plasma; Table S3: Accelerated stability in dog plasma; Table S4: Frozen PK sample stability in dog plasma and mouse plasma; Table S5: Freeze-thaw and autosampler stability for mouse plasma; Table S6: Freeze-thaw and autosampler stability for dog plasma.
