# Supplementary material for: Novel Approaches to Monitor Pharmacokinetics and Metabolism of Gemcitabine-Ibandronate Conjugate in Mice and Dogs
Source: Molecules. 2025 Jan 16;30(2):354. doi: 10.3390/molecules30020354 (PMC11767451; doi:10.3390/molecules30020354)

Supplementary Materials

Supplementary Tables

Table S1. Intra- and inter-run accuracy and imprecision

		Parameter	Mouse plasma range [%]	Dog plasma range [%]
GEM	Intra-run	%-Accuracy	86.4 - 110.8	91.8 - 106.2
		%-Imprecision	4.1 - 13.3	3.8 - 10.6
	Inter-run	%-Accuracy	95.1 - 106.6	97.1 - 100.9
		%-Imprecision	7.3 - 11.8	6 - 9.9
dFdU	Intra-run	%-Accuracy	92 - 108.2	92.2 - 108.9
		%-Imprecision	4.8 - 14.2	4.1 - 13.1
	Inter-run	%-Accuracy	96.7 - 106.4	97.8 - 107
		%-Imprecision	6.6 - 13.6	6.5 - 9.8
GEMMP	Intra-run	%-Accuracy	86.8 - 113.5	88.1 - 112.3
		%-Imprecision	3.3 - 15	5.8 - 14.9
	Inter-run	%-Accuracy	92.6 - 105	91.7 - 106.9
		%-Imprecision	8 - 13.9	9.9 - 12.6
dFdUMP	Intra-run	%-Accuracy	84.1 - 109.8	89.9 - 110.5
		%-Imprecision	3.7 - 14.5	4.2 - 13.2
	Inter-run	%-Accuracy	86.2 - 101.3	97.3 - 107.7
		%-Imprecision	7.0 - 11.0	5.1 - 11.2
IB	Intra-run	%-Accuracy	97.2 - 108.3	95.7 - 109.2
		%-Imprecision	0.7 - 9.2	1.4 - 13.1
	Inter-run	%-Accuracy	100.9 - 103.1	99.7 - 105.6
		%-Imprecision	2.6 - 8.7	4.1 - 10.3
GEM-IB	Intra-run	%-Accuracy	96.9 - 108.4	100.2 - 111.9
		%-Imprecision	1.7 - 10	3.8 - 12.9
	Inter-run	%-Accuracy	101.5 - 106.7	103 - 107.1
		%-Imprecision	5.4 - 6.9	6.9 - 9.5

Table S2. Accelerated stability in mouse plasma.

	4* hours RT		24 hours RT		4 hours 4°C		24 hours 4°C	
	%-ACC	%-SD	%-ACC	%-SD	%-ACC	%-SD	%-ACC	%-SD
GEM	89.3	29.2	91.0	25.0	94.5	23.1	87.9	29.8
dFdU	83.8	13.9	85.2	14.1	84.5	15.6	87.0	17.6
GEMMP	87.9	19.3	88.1	28.1	91.9	21.7	91.4	21.5
dFdUMP	80.5	15.2	81.5	16.0	83.0	23.6	81.9	21.2
IB*	106.4	7.4	101.7	2.3	97.0	6.6	99.7	4.0
GEMIB*	31.0	35.4	0.0	0.1	64.2	7.6	2.9	1.6

*__IB__ and **GEMIB** were tested for 2 hours and 24 hours at room temperature and 4°C. Abbreviations: %-ACC, percent mean accuracy; %-SD, percent standard deviation.

Table S3. Accelerated stability in dog plasma.

	4* hours RT		24 hours RT		4 hours 4°C		24 hours 4°C	
	%-ACC	%-SD	%-ACC	%-SD	%-ACC	%-SD	%-ACC	%-SD
GEM	101.7	12.3	95.4	13.7	102.7	10.5	94.8	10.7
dFdU	101.3	12.9	98.4	10.9	100.5	16.9	105.3	14.5
GEMMP	98.2	12.9	98.2	19.2	89.5	14.5	88.1	15.5
dFdUMP	86.7	17.7	80.5	12.4	84.2	18.5	85.2	18.6
IB*	101.0	1.6	118.5	4.5	99.3	2.2	108.8	16.0
GEM-IB*	83.5	5.0	13.7	1.2	99.3	8.9	83.6	11.5

*__IB__ and **GEM-IB** were tested for 2 hours and 24 hours at room temperature and 4°C. Abbreviations: %-ACC, percent mean accuracy; %-SD, percent standard deviation.

Table S4. Frozen PK sample stability in dog plasma and mouse plasma.

	Dog Plasma		Mouse Plasma	
	%-ACC	%-SD	%-ACC	%-SD
**GEM-IB***	105.5	40.4	111.4	38.0
**IB***	107.8	2.5	113.0	25.8
**GEM****	88.5	28.5	94.2	11.8
**GEMMP****	58.1	74.1	64.0	45.0
**dFdU****	63.4	76.4	71.5	33.4
**dFdUMP****	49.3	107.0	108.1	35.8

*__IB__ and **GEM-IB** were tested after 1.5 years storage at <-70°C. ****GEM**, **GEMMP**, **dFdU** and **dFdUMP** were tested after 2.5 years storage at <-70°C. Abbreviations: %-ACC, percent mean accuracy; %-SD, percent standard deviation.

Table S5. Freeze-thaw and autosampler stability for mouse plasma.

	3 Freeze-thaw cycles		24 hours Autosampler		48 hours Autosampler	
	%-ACC	%-SD	%-ACC	%-SD	%-ACC	%-SD
GEM	103.4	11.8	102.0	12.3	107.0	9.0
dFdU	109.5	17.4	103.9	12.3	103.4	10.3
GEMMP	92.4	16.8	96.9	15.3	100.4	12.0
dFdUMP	87.7	18.1	102.4	11.8	98.8	12.0
IB	94.5	6.9	97.6	3.0	98.6	1.7
GEM-IB	16.9	6.7	96.8	13.2	59.7	21.1

Abbreviations: %-ACC, % Accuracy; %-SD, % Standard deviation.

Table S6. Freeze-thaw and autosampler stability for dog plasma.

	3 Freeze-thaw cycles		24 hours Autosampler		48 hours Autosampler	
	%-ACC	%-SD	%-ACC	%-SD	%-ACC	%-SD
GEM	103.4	11.8	100.7	10.5	106.8	9.5
dFdU	109.5	17.4	99.0	14.4	102.0	16.4
GEMMP	92.4	16.8	92.4	14.4	90.0	14.8
dFdUMP	87.7	18.1	92.6	16.2	84.8	17.0
IB	106.5	2.9	111.2	4.9	107.7	1.8
GEM-IB	84.7	5.7	86.2	6.1	63.4	6.4

Abbreviations: %-ACC, % Accuracy; %-SD, % Standard deviation.

Supplementary Figures

Supplementary Figure Legends

Supplementary Figure S1

Representative calibration curves and representative extracted ion chromatograms. Figures S1.a and S1.b show calibration curves and representative extracted ion chromatograms for **IB** and **GEM-IB** in mouse plasma (S1.a) and dog plasma (S1.b). A and B show calibration curves for **IB** and **GEM-IB**, C and D show extracted ion chromatograms of blank extracted mouse samples for **IB** and **GEM-IB** (S1.a C and D) and dog plasma for **IB** and **GEM-IB** (S1.b C and D). The row below (E and F) shows calibrator samples at the lower limit of quantitation 40 ng/mL and 10 ng/mL for **IB** and **GEM-IB** respectively. H and G show representative extracted ion chromatograms of **IB** and **GEM-IB** in study samples after infusion with **GEM-IB**. Similarly, Figure S1.c A-B and Figure S1.d A-B show representative calibration curves for **GEM** (A) and **GEMMP** (B). The following 3 rows show blank extracted matrix (C and D), the lower limit of quantitation samples (E and F) and study samples for **GEM** and **GEMMP** (G and H) in mouse (S1.c C to H) and dog plasma (S1.d C to H). Finally, the **dFdU** and **dFdUMP** in mouse and dog plasma is displayed in Figure S1.e and S1.f, respectively. Figure S1.e A-B and Figure S1.f A-B show representative calibration curves for **dFdU** (A) and **dFdUMP** (B) and the following rows show blank extracted matrix (C and D), the lower limit of quantitation samples (E and F) and study samples for dFdU and **dFdUMP** (G and H) in mouse (S1.e C to H) and dog plasma (S1.f C to H).

Supplementary Figure S1.a

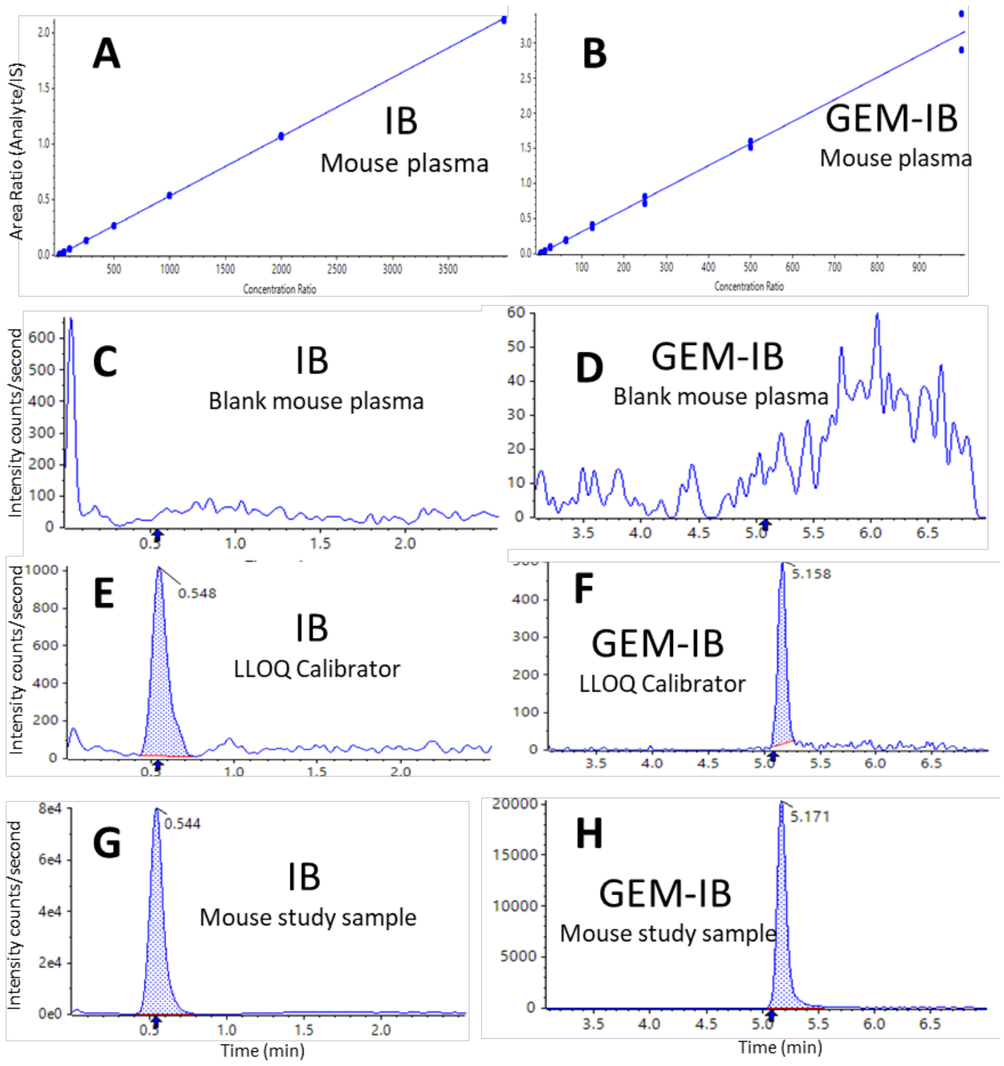

Supplementary Figure S1.b

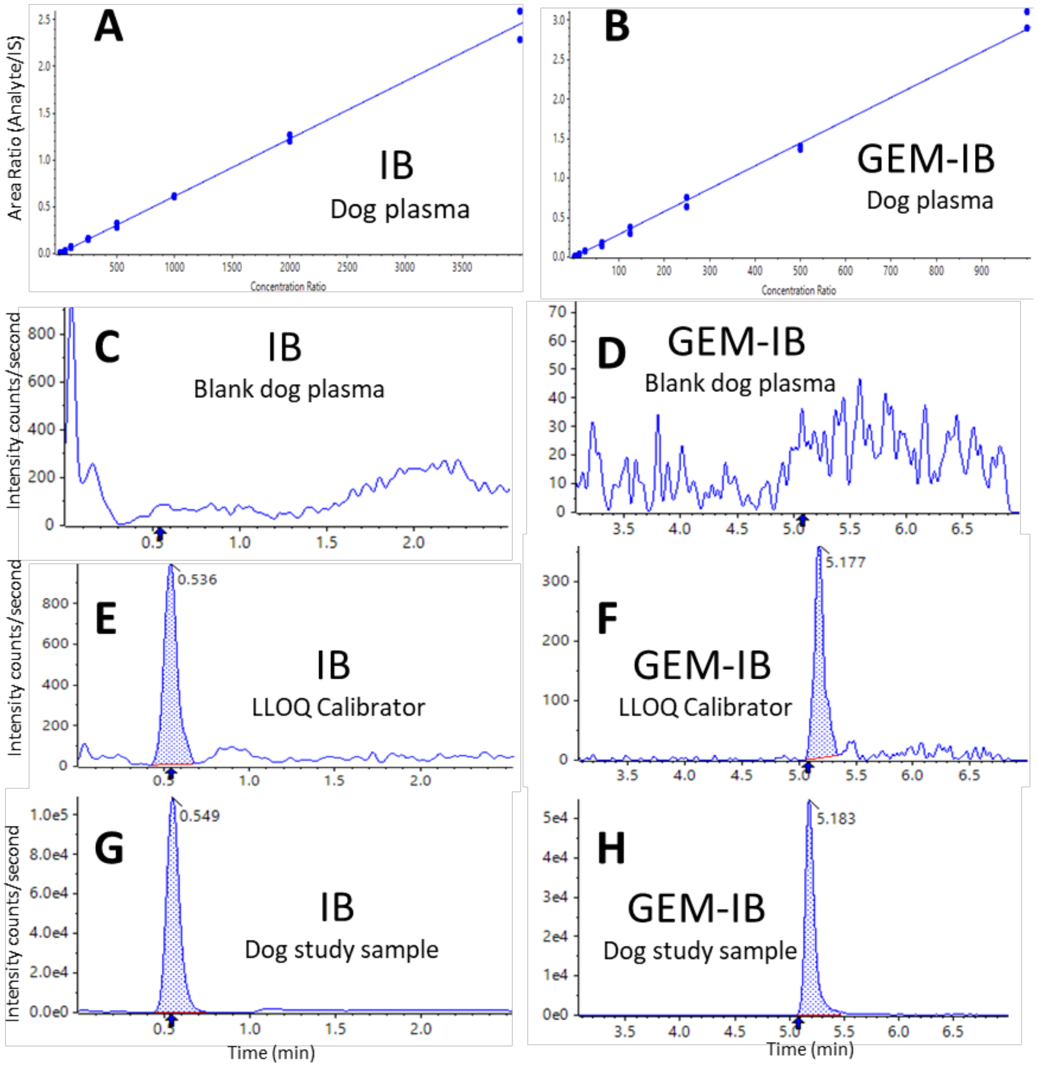

Supplementary Figure S1.c

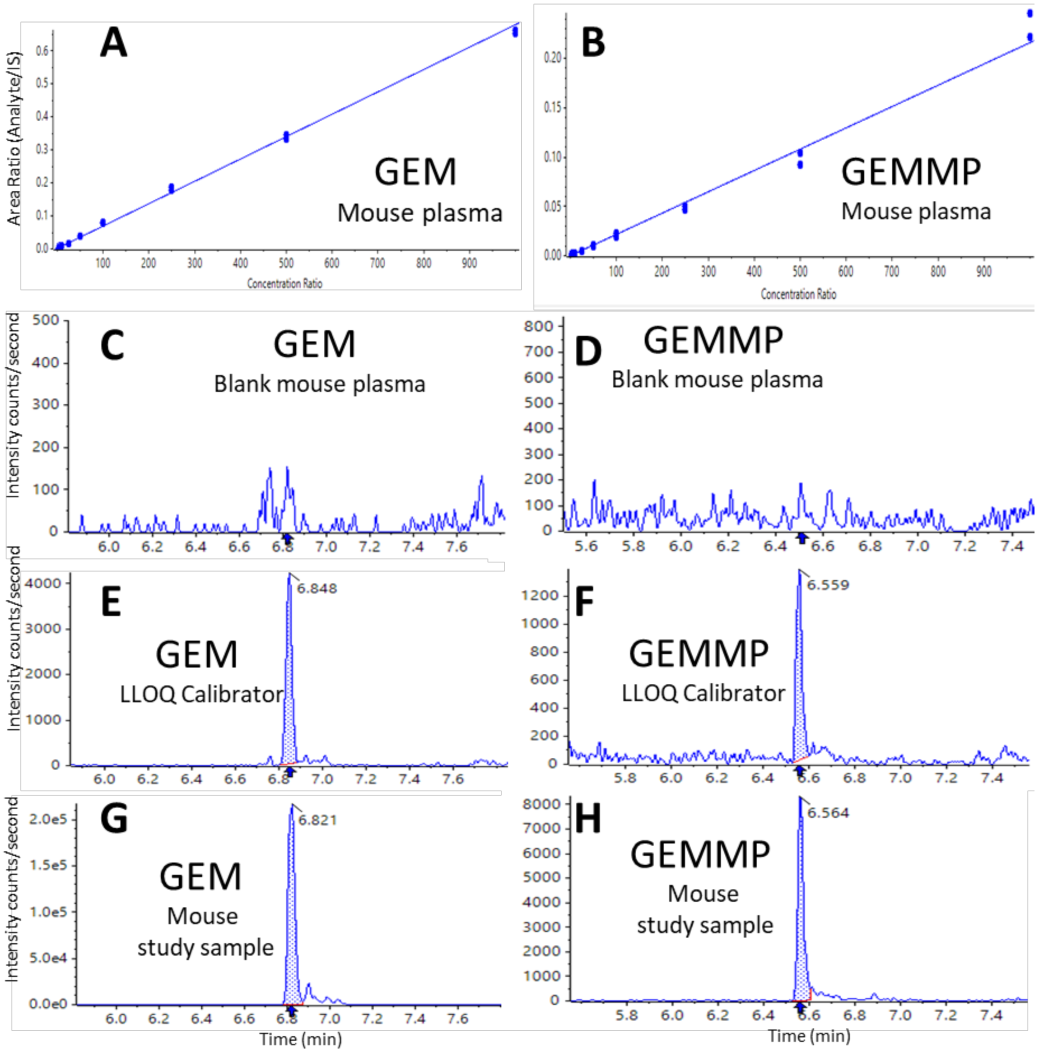

Supplementary Figure S1.d

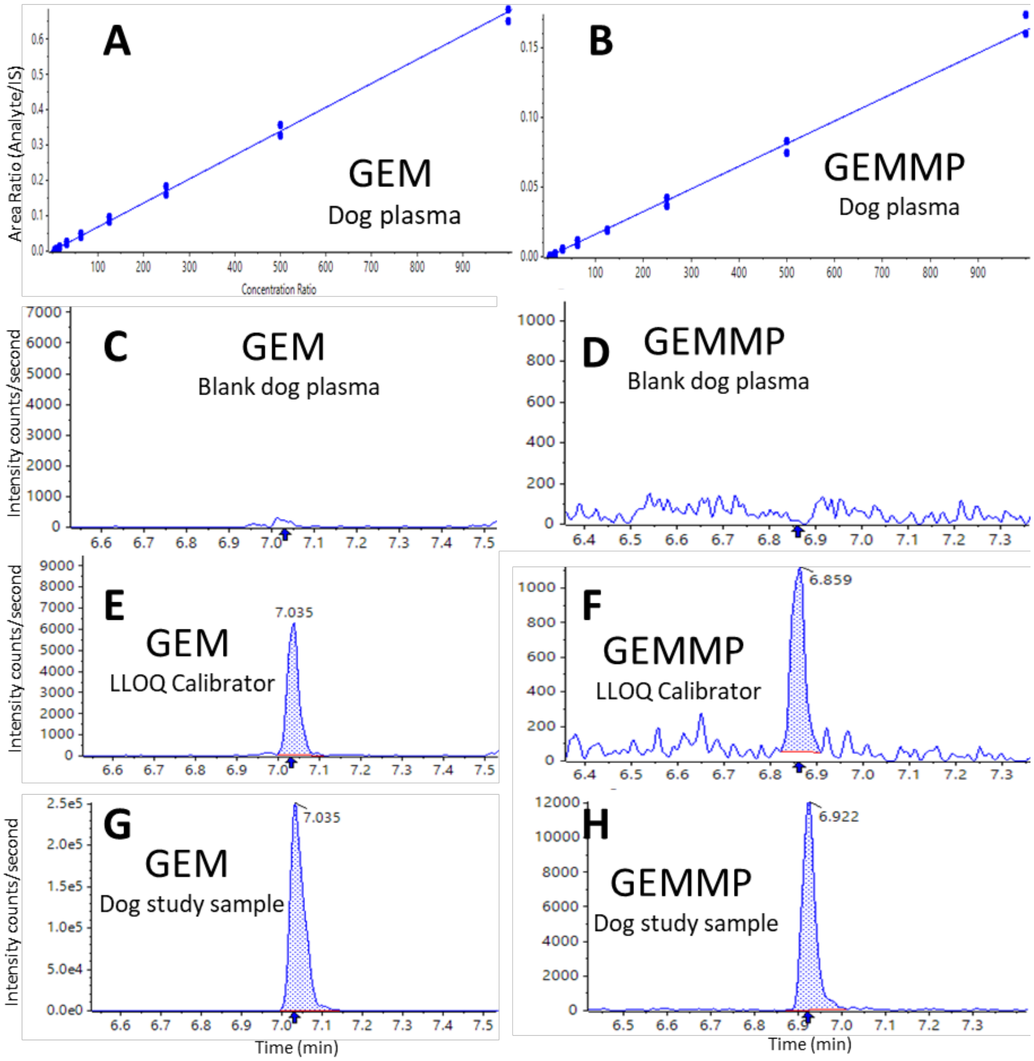

Supplementary Figure S1.e

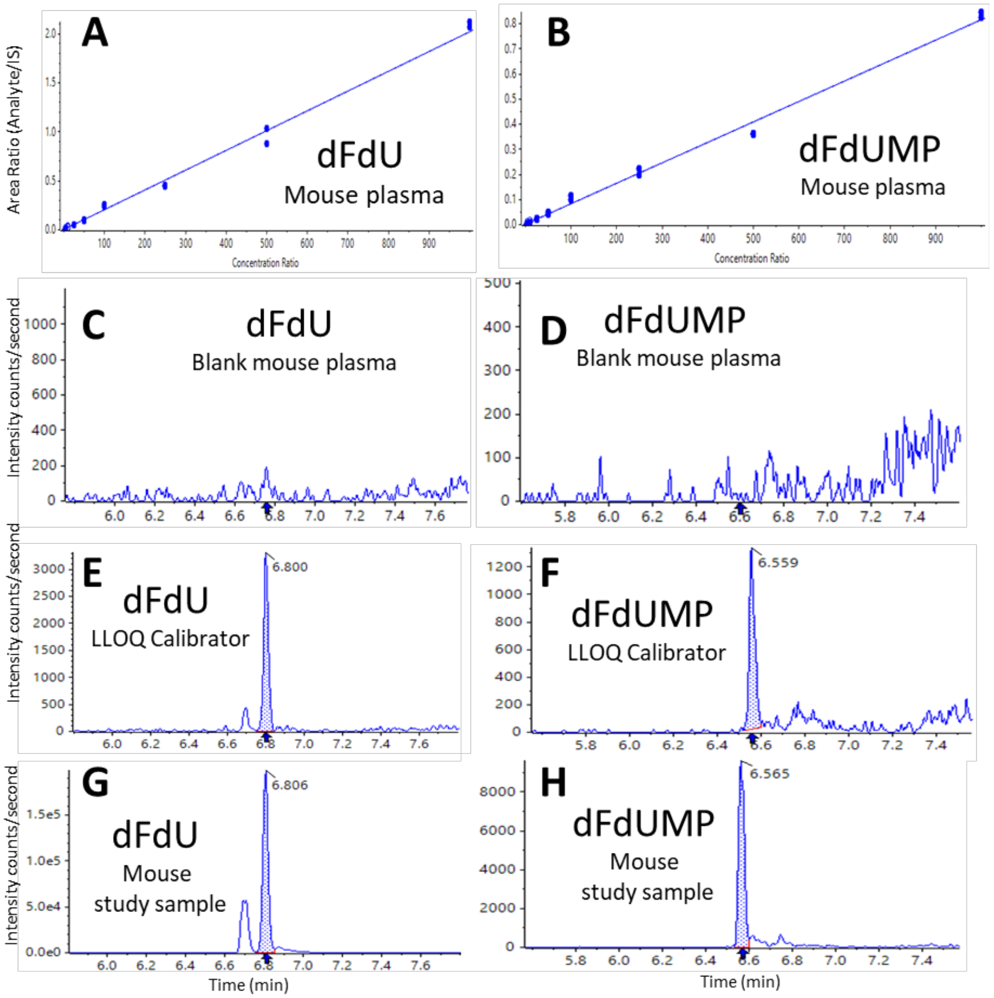

Supplementary Figure S1.f

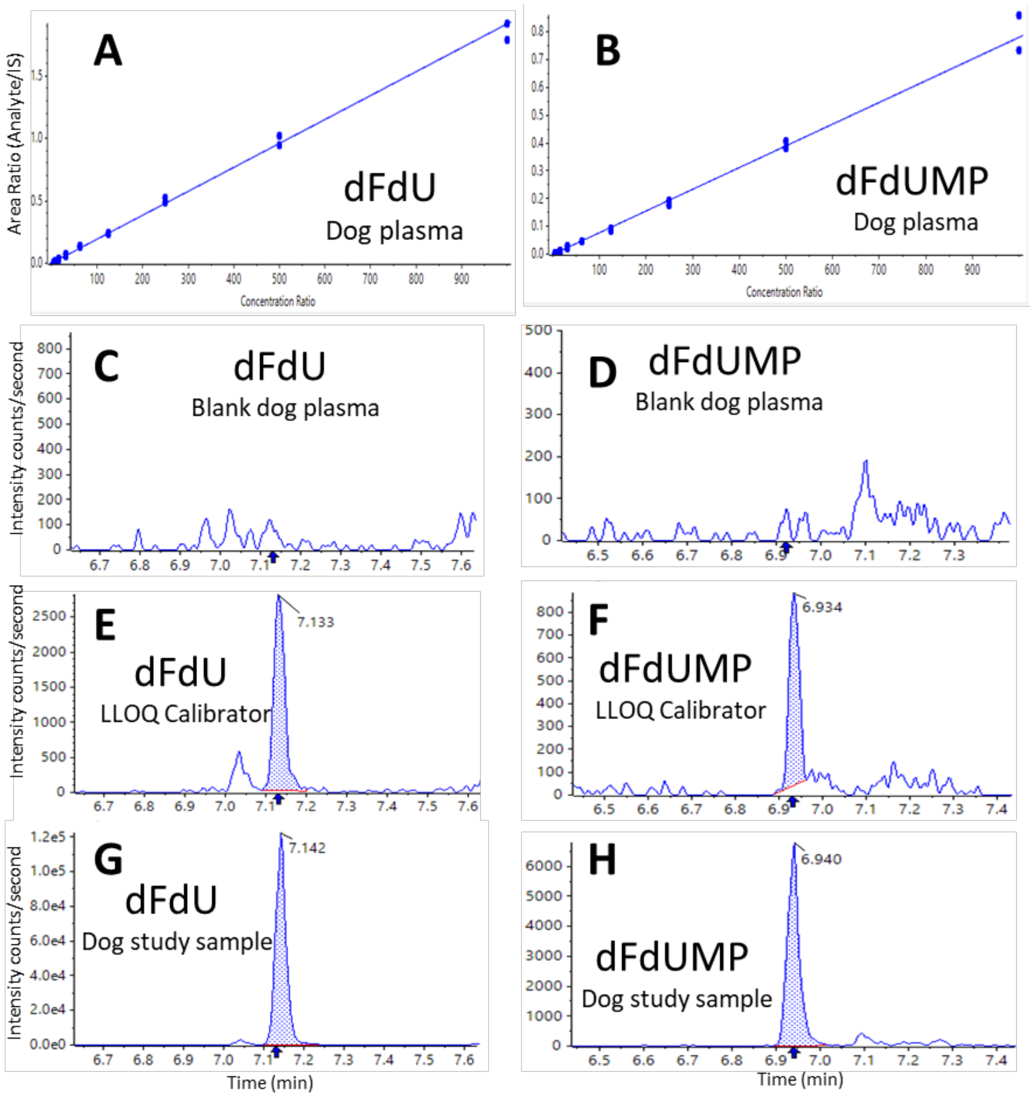

Supplement: Supplementary file 1 [file molecules-30-00354-s001.zip › molecules-3370400-supplementary.pdf]
